# Effects of Different Calcium Sources on Mechanical Properties of Metakaolin Geopolymers

**DOI:** 10.3390/ma17092087

**Published:** 2024-04-29

**Authors:** Yiren Wang, Jiangtao Zhang, Jie Liu, Deke Fan, Haiyang Qu, Lingzhu Zhou, Sen Zheng

**Affiliations:** 1 School of Environment and Civil Engineering, Dongguan University of Technology & Guangdong Provincial Key Laboratory of Intelligent Disaster Prevention and Emergency Technologies for Urban Lifeline Engineering, Dongguan 523808, China; wangyr@dgut.edu.cn (Y.W.); lingzhu_zhou@163.com (L.Z.); 2 China Building Materials Academy, CNBM Zhongyan Technology Co., Ltd., Beijing 100024, China; deco2004@163.com (D.F.); juhy1993@163.com (H.Q.); 3 School of Civil Engineering, Sun Yat-sen University, Zhuhai 519082, China; j.liu.post@outlook.com; 4 ENAC/IIC/LHE, Ecole Polytechnique Fédérale de Lausanne, 1015 Lausanne, Switzerland; sen.zheng@epfl.ch

**Keywords:** geopolymer, calcium sources, mechanical properties, hydration behavior

## Abstract

Metakaolin-based geopolymers have substantial potential as replacements for cement, but their relatively inferior mechanical properties restrict their application. This paper aims to enhance the mechanical properties of metakaolin-based geopolymers by incorporating appropriate amounts of calcium sources. CaCO_3_, Ca(OH)_2_, and CaSO_4_ are three types of calcium sources commonly found in nature and are widely present in various industrial wastes. Thus, the effects of these three calcium sources on the performance of metakaolin-based geopolymers were studied. Through the analysis of the mechanical properties, heat-release behavior during hydration, hydration products, and microstructure of geopolymers, the effectiveness of the aforementioned calcium sources in improving the performance of metakaolin-based geopolymer was evaluated, and the mechanisms of action were elucidated. The results indicate that the pozzolanic reaction between CH and MK could promote MK hydration and increase the proportion of CASH gel in the hydration products, thereby facilitating the setting of the geopolymer and enhancing its strength. CS could react with the active aluminates in MK to form ettringite, thus forming a higher early strength. CC had a lower reactivity with MK and does not improve the performance of MK-based geopolymers.

## 1. Introduction

Ordinary Portland cement (OPC) is the most commonly used building material, and its production is responsible for approximately 8–9% of total CO_2_ emissions due to the burning of high-quality coal and the decomposition of limestone [1,2]. There are reports declaring that the emission of CO_2_ into the atmosphere from cement production should be stopped completely to stop global warming [3]. Benefiting from the low carbon footprint and good mechanical properties of geopolymers, they are considered to be a potential alternative to cement [4,5,6]. A typical geopolymer is formed by a precursor (blast furnace slag, fly ash metakaolin) and an alkali solution (NaOH, KOH, Na_2_SiO_3_, and K_2_SiO_3_) [7]. Furthermore, the geopolymers are distinguished into two systems based on the calcium content of the precursors, including geopolymers derived from low-calcium content such as metakaolin, and cement hydrates formed from high-calcium content such as blast furnace slag [8]. For low-calcium systems, their main hydration products are 3D network-type sodium/potassium aluminosilicate hydrate gels (Na (K)-A-S-H, depending on the alkali activator used) [9,10], while for high-calcium system, their main hydrate products are chain-type calcium silicate hydration (C-A-S-H) gels [11]. It is reported that compared with N-A-S-H gel, C-A-S-H gel is more favorable for paste densification and improvement in the mechanical properties of composites [12,13,14], and thus alkali-activated slag has received relatively wide attention [6,15]. However, blast furnace slag, as a highly reactive silica-aluminate, is also extensively used as a supplementary cementitious material in cement production [16]. This leads to a scarcity of available high-calcium precursors. Therefore, more high-calcium precursors must be developed.

Several studies have shown that those two gels, N-A-S-H and C-A-S-H gel, can coexist in geopolymers [14,17,18,19]. Furthermore, N-A-S-H gel may be transformed into C-A-S-H gel with the increase in calcium content in precursors [14,17,18,20]. Li et al. [20] mixed metakaolin, slag, and silica fume in proportion to prepare precursors with different calcium content and added a mixed solution of water glass and NaOH to prepare a geopolymer. Their results show that the transformation of N-A-S-H gel into C-(A)-S-H gel is promoted, resulting in a more compact structure of the geopolymer. However, it is noteworthy that slag is the only calcium source in their research, and thus it is difficult to distinguish the role of slag and CaO. Granizo et al. [17] discussed the influence of Ca(OH)_2_ on alkali-activated metakaolin and found that a small amount of C-S-H gel is formed when Ca(OH)_2_ is present. Similarly, Dombrowski et al. [21] studied the influence of calcium content on the fly-ash-based geopolymer. They observe the presence of C-S-H and zeolite phases in samples with high calcium content. Yip et al. [18] investigated the role of seven different calcium silicate materials in geopolymer. According to their results, in addition to the crystallinity of the calcium source, the alkalinity of the alkali solution used is also critical to the hydration of the geopolymer. At low alkalinities, the rapid release of calcium from artificial calcium silicate promotes C-S-H formation, while natural calcium silicate releases less calcium, and thus forms little C-S-H. At high alkalinities, the hydration products of geopolymers are always dominated by N-A-S-H gel, regardless of the calcium silicate source, which has little effect on the hydration products. The above research shows that when a calcium source is introduced to improve the geopolymer, its hydration reaction seems to become more complex and the factors affecting hydration are also increased. The conversion of N-A-S-H and C-A-S-H in different conditions needs to be further clarified. Furthermore, another noteworthy question is that the continued use of artificial or natural calcium sources is inappropriate, since the use of geopolymers is intended to reduce or even avoid the consumption of this type of material. Therefore, it is important to find more low-carbon calcium sources and to further discuss their impact on geopolymers.

In fact, there are many high-calcium industrial solid wastes that are good calcium sources for geopolymers, and we have simply divided them into three classes in this study. Class I: Ca(OH)_2_ is a major phase composition, with the most representative being calcium carbide residue (CCR). CCR, a by-product from the acetylene production, is mainly composed of Ca(OH)_2_ [22]. It has been previously demonstrated that CCR can be used as an alternative to Ca(OH)_2_ [22,23,24,25,26]. Class II: CaCO_3_ is major phase composition, and soda residue (SR) is chosen as a representative in this study. SR is a by-product from the Solvay process [27]. Its components are relatively complex, mainly including CaCO_3_, CaCl_2_, CaSO_4_, and Ca(OH)_2_ [26,27,28]. Zhao et al. [28] reported that the addition of SR in appropriate amounts could provide calcium ions to the fly-ash-based geopolymer, resulting in the coexistence of C-S-H, C-A-S-H, and N-A-S-H gel, thus enabling a denser microstructure of the samples. Nevertheless, Qing et al. [26] contended that SR appears to have a less positive effect on the hydration process because the calcium ions involved in geopolymerization are provided by a small amount of Ca(OH)_2_ in SR rather than CaCO_3_. Class III: CaSO_4_ is major phase composition, with the most representative being gypsum. Gypsum (CaSO_4_·2H_2_O) is a prominent building material, which is also an excellent calcium source. In recent years, to reduce the consumption of natural gypsum, a lot of research has been devoted to the replacement of natural gypsum with phosphogypsum (PG) or flue gas desulfurization gypsum (FGDG) for the production of cement and geopolymers [29,30,31,32,33,34]. However, when using gypsum, the influence of SO_4_^2−^ is usually greater than that of calcium ions because of the formation of ettringite [33]. It is evident from the above literature that regardless of the calcium source, the role of calcium ions is always similar, that is, to promote the conversion of N-A-S-H to C-A-S-H. In contrast, the role of the anion is more obvious and different. For example, carbonates accelerate the carbonation of geopolymers due to the presence of CO_3_^2−^ [26], while sulfates lead to the formation of ettringite because of the presence of SO_4_^2−^ [33]. However, these findings are independent and a comprehensive comparative study discussing the role of different anions in geopolymers is lacking. Therefore, the role of different calcium sources in geopolymers needs to be further discussed.

Another problem with the use of those solid waste is that the composition of the solid waste is complex, and the property of the material may vary unpredictably due to the change in impurities. Complex composition is the key to prevent the effective use of solid waste resources. Thus, the identification of the key ions of high-calcium solid waste that plays a major role in geopolymers and their mechanisms of action is crucial for the better use of high-calcium solid waste in geopolymers. Although it is difficult to determine the effect of each impurity on the geopolymer due to the high number of impurities, it is feasible to determine the effect of relatively high levels of impurities on it.

To address the above issues, the following two points were focused upon in this study. First, the effects of three calcium sources, including Ca(OH)_2_, CaCO_3_, and CaSO_4_, on the metakaolin-based geopolymers were identified using chemical reagents. After that, these samples were compared with samples prepared from Ca(OH)_2_ replaced by CCR, CaCO_3_ replaced by SR, and CaSO_4_ replaced by PG and FGDG, respectively. This comparison was used to identify the key components of solid waste that affect geopolymers. Second, the pure material of the key component was mixed with the metakaolin-based geopolymers to confirm its effect, and to elucidate the mechanism of influence. The above is intended to provide new insights into the application of high-calcium solid wastes in geopolymers.

## 2. Materials and Methods

### 2.1. Materials

**Figure 1:**
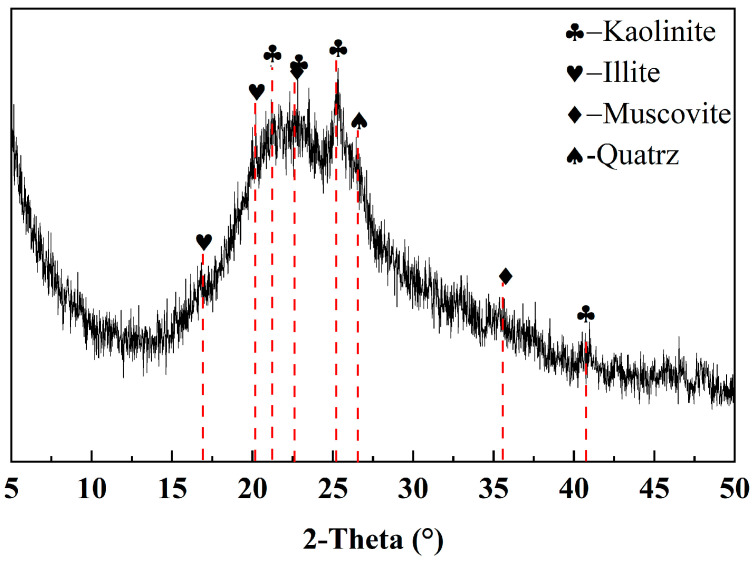
XRD patterns of MK.

**Figure 2:**
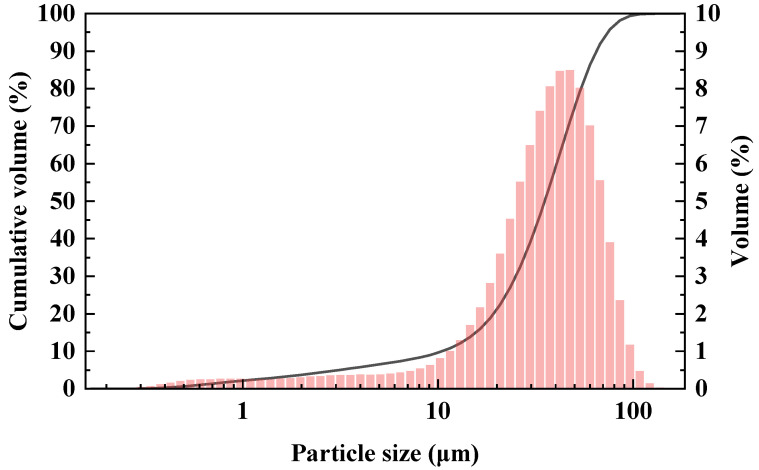
Particle size distribution curves of MK.

The metakaolin used in this study was sourced from a chemical company (Shanxi, China). The chemical composition and mineral composition of metakaolin (MK) are presented in Table 1 and Figure 1, respectively. It can be observed that the primary chemical components in MK were SiO_2_ (55.54%) and Al_2_O_3_ (40.61%). From the perspective of mineral composition, MK was predominantly amorphous, while also containing minor amounts of kaolinite, illite, muscovite, and quartz impurities. The particle size distribution of MK is shown in Figure 2. NaOH (Analytical Reagent, shortened AR, 98%), Ca(OH)_2_ (AR, 95%), CaCO_3_ (AR, 98%), and CaSO_4_ (AR, 97%) were purchased from Zhiyuan Chemical Reagent Co., Ltd. (Tianjin, China).

### 2.2. Experiments

#### 2.2.1. Sample Preparation

The primary difference between different calcium sources is the anion type. It is important to investigate the effect of several common calcium-containing compounds on geopolymers to determine the impact of different anions on the performance of geopolymers. Therefore, the geopolymers were first prepared using three chemical reagents, namely Ca(OH)_2_ (CH), CaCO_3_ (CC), and CaSO_4_ (CS), mixed with MK based on 2 mol NaOH solution as an alkali activator and a water–solid ratio of 0.5. After that, the chemical reagent was replaced with the corresponding solid waste and compared. Detailed ratios are shown in Table 2.

The 2 mol NaOH solution was prepared and left to cool to room temperature (approximately 24 h) before sample preparation. The calcium source and metakaolin were mixed in a blender for 3 min to ensure that homogeneous precursors were obtained. The prepared NaOH solution was then added to the precursor according to the designed water–solid ratio. The solution and precursors were mixed in the blender for 5 min to obtain a uniform paste. Finally, the prepared pastes were poured into cylindrical plastic molds with 50 mm in diameter and 50 mm in height, cured at room temperature for 24 h, then demolded, and stored in a curing room with 20 ± 2 °C and relative humidity ≥95% for 7 and 28 d.

#### 2.2.2. Characterization of the Geopolymers

The unconfined compressive strength (UCS) was measured by a Humboldt HM-5030 loading machine (Humboldt, Elgin, IL, USA) with a rate of 1 mm/min. Three tests were carried out for each group, and the average value was determined to be the definitive UCS for the samples.

The setting times of the samples were determined using a Vicat apparatus following the ASTM C191 standard test method [35]. Only the final setting time was measured due to the high viscosity of the samples.

After the UCS tests, the fragments of the samples were collected and grinded into powder. The powder was added into deionized water according to a liquid–solid ratio of 10 and stirred for 5 min at 180 rpm using a magnetic stirrer. After the solution was filtered, its pH value was measured with a pH meter (0.01 accuracy).

The hydration heat evolution of the fresh geopolymers in 72 h was recorded by an isothermal calorimeter isothermal calorimetry instrument (TAM AIR Calorimeter).

After the UCS tests, the fragments of the samples were collected. Part of the fragments was grinded into powder (<75 μm), immersed into isopropanol (AR, 99.9% purity) for 24 h, and dried at 40 °C in a drying oven for 24 h to stop the hydration for subsequent characterization. The other fragments were directly immersed in isopropanol and then dried to stop the hydration for further characterization.

X-ray diffraction (XRD) patterns of powder samples were collected from 5° to 50° 2θ with the scanning speed 5°/min. Thermogravimetric (TG) and derivative thermogravimetric (DTG) analyses were performed with a TG 209 F3 Nevio thermogravimetric analyzer (Netzsch, Selb, Germany). The curves were obtained by heating 15 mg of the powder sample from 30 °C to 900 °C at a rate of 10 °C/min under a N_2_ atmosphere. Fourier transform infrared spectroscopy (FTIR) spectra of powder samples were obtained using a Nexus 670 infrared spectrometer (Thermo, Wilmington, NC, USA) in the range of 4000 to 400 cm^−1^. The transmission electronic microscope (TEM) image and energy dispersive X-ray spectra collection was performed using JEM-2100F (JEOL, Tokyo, Japan) and Ultim Max 80 (Oxford Instruments, Oxford, UK), respectively, to study the microstructure of samples at 28 d.

## 3. Results

### 3.1. Effects of Calcium Sources on Final Setting Time

Table 3 shows the final setting time of the metakaolin geopolymers. As can be seen, the calcium source has a significant effect on the setting time of metakaolin geopolymers, ranging from 20 to 600 min. The final setting time of the plain MK paste is approximately 358 min as the control group. The addition of CC causes the final setting time of the geopolymer paste to nearly double to 604 min, whereas the addition of CH accelerates the setting of the geopolymer paste. This accelerated setting effect is significant, reducing the final setting time of the MK-CH paste by a factor of approximately 15 compared to the plain MK paste. Similarly, the addition of CS also reduces the final setting time of the geopolymer pastes. As shown in Table 3, the final setting time of MK-CS paste is reduced by 221 min compared to the plain MK paste, but presents a lesser reduction magnitude compared to MK-CH. The final setting time of MK and MK-CS is acceptable during construction, while too long a final setting time of MK-CC and too short a final setting time of MK-CH are considered to be unfavorable to construction.

### 3.2. Effects of Calcium Sources on Mechanical Properties

**Figure 3:**
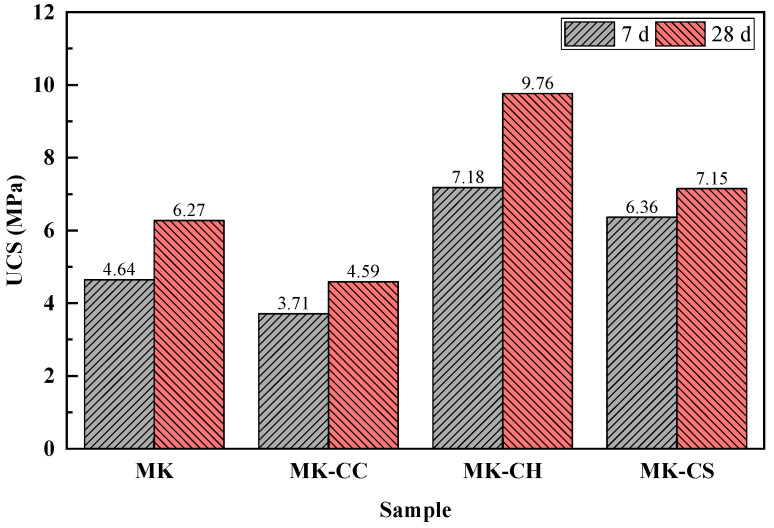
UCS results of the metakaolin geopolymers.

Figure 3 shows the UCS results of the metakaolin geopolymers at curing age of 7 d and 28 d. It can be observed that the addition of CH and CS effectively enhanced the UCS of the geopolymer, with MK-CH and MK-CS showing an increase in UCS by 54.74% and 37.07%, respectively, at 7 d and by 55.66% and 14.04%, respectively, at 28 d. In contrast, the addition of CC reduced the UCS of the geopolymer, with MK-CC’s UCS at 7 d and 28 d, decreasing by 20.04% and 26.79%, respectively, compared to MK. Furthermore, the strength development over time of the geopolymers with different calcium sources was distinctly different. From 7 to 28 d, the UCS of MK, MK-CC, MK-CH, and MK-CS increased by 35.13, 23.72, 35.93, and 12.42%, respectively. MK-CC not only demonstrated lower UCS but also exhibited a smaller rate of strength growth over time compared to MK. While MK-CS possessed a relatively higher UCS, it experienced the least increase in strength rate. The strength growth rates of MK and MK-CH were virtually identical.

This strength development behavior may be related to the effect of anionic in different calcium sources. The addition of CH provided additional OH^-^, which could promote the pozzolanic reaction of metakaolin, enhancing the reaction rate of metakaolin, thereby significantly improving the early strength of the geopolymer [36,37]. At the same time, the additional Ca^2+^ in CH could increase the Ca/Si ratio of the C(A)SH gel, benefiting the strength development of the geopolymer [38]. The addition of CS provided additional SO_4_^2−^, which might have facilitated the formation of ettringite [39]. This is key to the higher early strength exhibited by geopolymers containing CS. However, CS cannot directly react with metakaolin, and thus as the curing time increased, the rate of strength growth was relatively smaller. The addition of CC also failed to promote the hydration of MK, and the CO_3_^2−^ did not substantially aid in the formation of hydration products within geopolymers, resulting in the minimal strength growth.

### 3.3. Effects of Calcium Sources on pH of Metakaolin Geopolymers’ Pore Solution

**Figure 4:**
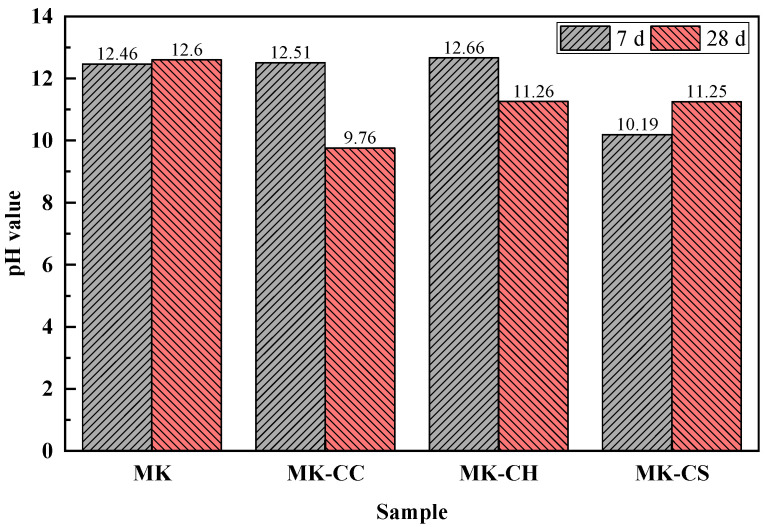
pH value of the metakaolin geopolymers.

Figure 4 displays the pH value of the pore solution in the metakaolin geopolymers at a curing age of 7 d and 28 d. The pH value of the pore solution in the plain MK sample remains approximately 12.5 with the increase in curing time. The addition of calcium sources, including CC and CH, has little effect on the pH value in the samples at 7 d, except for CS. As can be seen, the pH value of the pore solution in MK-CC and MK-CH samples is comparable to that in the MK samples at 7 d, with all hovering at approximately 12.5. In contrast, the pH value of the pore solution in the MK-CS samples decreased by 2.27, compared to the MK samples at 7 d, to 10.2. Generally, a higher pH value is more favorable for the dissolution and polycondensation of metakaolin. With the curing time increasing to 28 d, the pH value of the pore solution in MK-CC and MK-CH samples decreases obviously, especially the MK-CC samples, where the pH of the pore solution decreases from 12.51 to 9.76. Interestingly, the pH value of the pore solution in MK-CS samples increases instead, by approximately 1. The pH value of the pore solution could, to some extent, explain the strength development behavior of geopolymers [24]. The pore solution pH values of MK and MK-CH remained relatively high, ensuring the continuous dissolution and hydration of metakaolin particles. In contrast, the pH values for MK-CC and MK-CS were relatively lower, especially for MK-CC, where the pore solution pH value dropped below 10 after 28 d, which is not conducive to the hydration of metakaolin. Hence, it is evident that Ca(OH)₂ is the premium calcium source for enhancing the performance of metakaolin geopolymers.

### 3.4. Effects of Calcium Sources on Hydration Kinetics of Metakaolin Geopolymers

**Figure 5:**
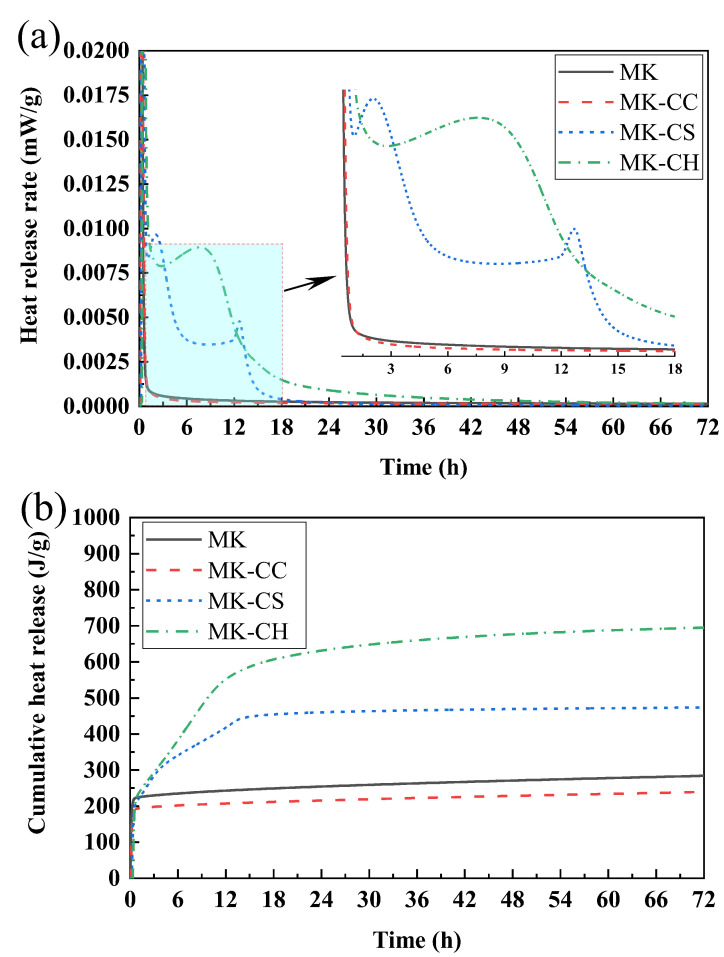
Heat release of the metakaolin geopolymers: (**a**) heat-release rate; (**b**) cumulative heat release.

Figure 5 show the effects of different calcium source on the heat-release rate (Figure 5a) and the cumulative heat release (Figure 5a) of metakaolin geopolymers. As shown in Figure 5a, plain MK paste shows a very weak exothermic peak of approximately 0.0004 mW/g at 3–9 h. After replacing 20% of MK with CC, this value is decreased by a quarter to approximately 0.0003 mW/g. It can be seen that the addition of CC has little effect on the heat-release-rate curve of MK pastes. However, the addition of CH and CS leads to a significant change in the heat-release-rate curve of MK pastes. After replacing 20% of MK with CH, a strong exothermic peak can be observed within 3–12 h, and the peak value is much higher than that of plain MK and MK-CC pastes, which is approximately 0.0085 mW/g. The most interesting thing is the heat-release-rate curve of MK-CS pastes, where two small exothermic peaks can be observed instead of one large exothermic peak. The first exothermic peak occurs within 1.5–3 h, and has a peak value of approximately 0.01 mW/g, while the second peak appears later, within 12–13.5 h, and has a peak value of approximately 0.005 mW/g.

As shown in Figure 5b, plain MK paste shows a cumulative heat release of 284 J/g at 72. As can be seen, the MK paste exhibits a large amount of exothermic behavior due to the dissolution of MK particles in the first 1 h, and the cumulative heat release in this stage is as high as approximately 225 J/g. After that, it does not exhibit significant exothermic behavior and continues to exotherm at a very low rate, and the cumulative heat release is only approximately 61 J/g in the remaining 71 h. The cumulative exothermic behavior of MK-CC paste is similar to that of plain MK paste, but it shows a lower cumulative heat-release value, which is approximately 240 J/g. Specifically, compared with plain MK paste, the cumulative heat release of the MK-CC paste is decreased by 20 J/g for both the dissolution stage and after dissolution, which are approximately 200 J/g and 40 J/g, respectively. The cumulative heat release of MK-CH paste is the highest, which is 695 J/g. In the dissolution stage (first 1 h), the cumulative heat release of MK-CH paste is similar to plain MK paste, which is also approximately 225 J/g. The second exothermic stage is then entered until the 12th h, during which the cumulative heat release increases by approximately 327 J/g, and the other 143 J/g is released during the remaining 60 h. The exothermic process of MK-CS paste is more complicated, and its cumulative heat release is approximately 473 J/g. The cumulative heat release in the dissolution stage is approximately 210 J/g, then increases by 100 J/g in 1–4 h, increases again by 130 J/g in 4–13.5 h, and is finally released 34 J/g in the remaining 58.5 h.

The above results show that different calcium sources have different effects on the early hydration process of MK paste. The addition of CC reduces the hydration rate and cumulative heat release of MK paste, which may indicate that CC is not conducive to the early hydration of MK. The addition of CH significantly increased the hydration rate and the cumulative heat release of the MK paste, which may suggest that more hydration products may be formed. The addition of CS may change the hydration process of MK paste, not only because the cumulative heat-release changes, but because new exothermic peaks also appear. This may indicate that the addition of CS changes the hydration products of metakaolin geopolymers.

### 3.5. Effects of Calcium Sources on Hydration Products Type

**Figure 6:**
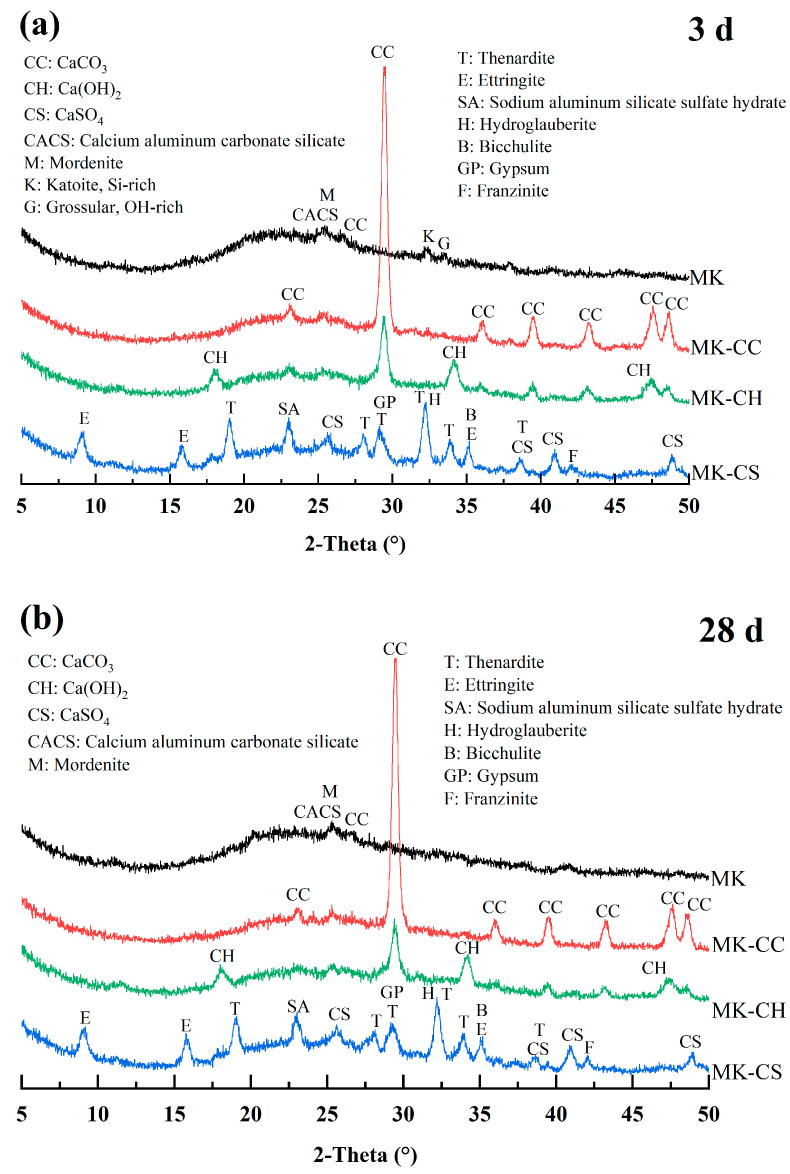
XRD patterns of the metakaolin geopolymers: (**a**) at curing age of 3 d; (**b**) at curing age of 28 d.

Figure 6 show the XRD patterns of the metakaolin geopolymers with different calcium sources at the curing age of 3 d (Figure 6a) and 28 d (Figure 6b), respectively. As can been seen, different calcium sources have significant influences on the hydration products of metakaolin geopolymers, especially concerning the types of hydration products formed.

The diffraction peak intensity of hydration products of plain MK samples is relatively weak at any curing age, but some crystalline phases, such as mordenite (M), calcium aluminum carbonate silicate (CACS), CaCO_3_ (CC), katoite (Si-rich, K), and grossular (OH-rich, G), can still be detected [40,41,42,43]. In addition, a large number of strong CC diffraction peaks can be seen in MK-CC samples. In MK-CH samples, both CC and CH diffraction peaks can be detected. The hydration products of MK-CS samples are the most complex, containing various types of hydration products, such as ettringite (E), sodium aluminum silicate sulfate hydrate (SA), thenardite (T), hydroglauberite (H), bicchulite (B), gypsum (GP), and franzinite (F) [44,45,46,47,48]. This is consistent with our previous speculation that the use of CaSO_4_ as a calcium source leads to the formation of a large number of new hydration products.

Furthermore, compared to 3 d of curing, no new diffraction peaks appear in the samples after 28 d of curing. This indicates that the curing time has little effect on the type of hydration product within 28 d. Meanwhile, the diffraction peak intensities of most of the hydration products do not change significantly after 28 d of curing, which indicate that the hydration products have higher crystallinity at 3 d. This may reflect that the samples may have been fully hydrated at 3 d.

**Figure 7:**
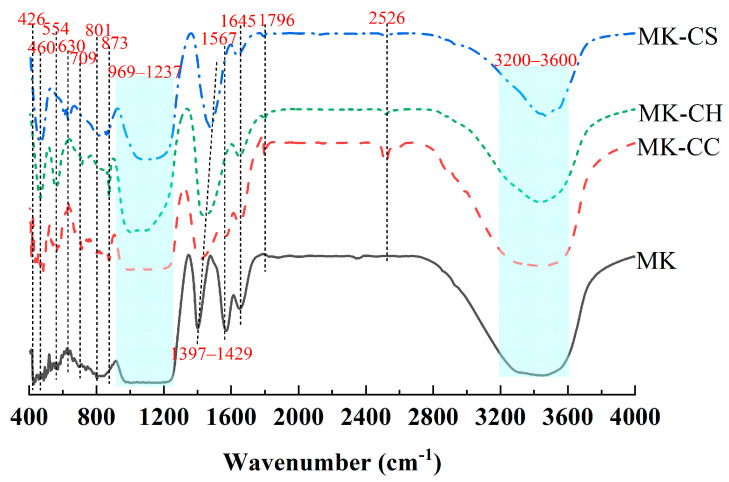
FITR spectra of the metakaolin geopolymers at curing age of 28 d.

The FTIR spectra of the hardened pastes are shown In Figure 7. The bonds at 3200–3600, 1645, and 1567 cm^−1^ were assigned to represented symmetric bending vibrations of H–O–H, caused by NASH gel and water [49]. The bonds at 2526 and 1796 cm^−1^ were attributed to CO_3_^2−^ vibrations in CaCO_3_, and hence, the related peaks were observed only in MK-CC [50]. The bonds at 969–1237 cm^−1^ might be due to an overlap of Si–O (CSH gels) and O–H bonds [49,50]. The bond at 801 cm^−1^ related to amorphous silica [51]. The bond at 630 cm^−1^ can be assigned to SO_4_^2−^, observed only in MK-CS [52]. The bond at 554 cm^−1^ was attributed to the Si–O–Al of MK [53]. The bonds at 460 and 426 cm^−1^ were asigned to the Si–O–Si vibration of CSH [54].

As shown in Figure 7, the peak area within 3200–3600 cm^−1^ was the largest for MK, indicating the highest content of water molecules in MK. Meanwhile, due to the extensive coverage area, the characteristic absorption bond of CH was not observed in MK-CH. For MK-CC, the characteristic bonds of CO_3_^2−^ at 2526 and 1796 cm^−1^ indicate that a significant amount of CC did not participate in the hydration reaction. While these bonds in MK-CH suggest that the metakaolin geopolymers underwent carbonation, resulting in products such as CC. No obvious carbonation products were observed in MK and MK-CS, possibly due to the lack of free Ca^2+^ reacting with CO_2_. The characteristic bond at 1567 cm^−1^, indicative of unbound water, is observed only in MK and MK-CC, suggesting that the hydration reaction of MK did not completely consume the water. The addition of CH and CS promoted the consumption of water by the geopolymer. Of which, CH primarily enhanced the pozzolanic reaction of MK, thereby increasing water consumption, and CS led to the formation of ettringite with higher bound water content, which also increased water consumption.

### 3.6. Effects of Calcium Sources on Hydration Products Content

**Figure 8:**
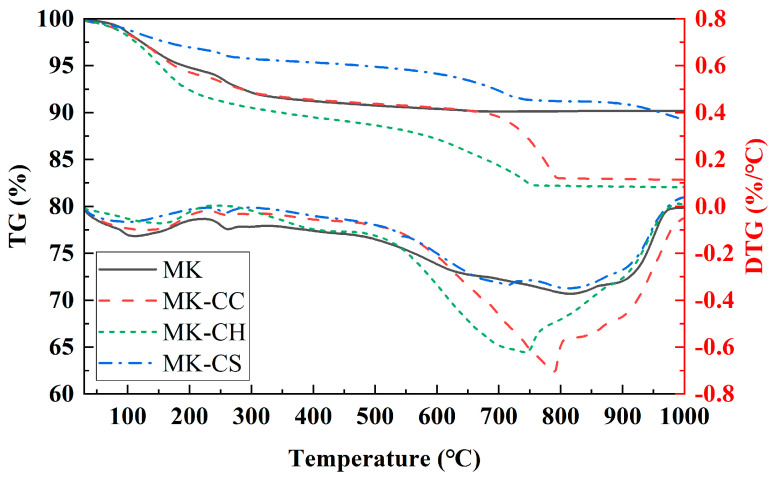
TG/DTG curves of the metakaolin geopolymers at curing age of 28 d.

Figure 8 shows the TG/DTG curves of hardened pastes after curing of 28 d. The mass loss in the samples mainly occurred in two different stages. The first stage was within 0–200 °C and was caused by the dehydration of NASH gel and ettringite [55,56,57]. The second stage was within 500–950 °C and was associated with the decomposed of CaCO_3_ and type Ⅰ CSH gel [58,59].

It can be observed that MK showed the greatest mass loss below 200 °C, with MK-CC also showing relatively high mass loss at this stage, while MK-CH and MK-CS had smaller mass losses. This indicates that the mass loss below 200 °C was primarily due to the decomposition of NASH, with a smaller proportion of mass loss caused by the decomposition of CSH and ettringite. The mass loss between 500–950 °C, due to the decarbonation reaction involving CC, was greatest in MK-CC, and MK-CH also experienced significant mass loss due to carbonation reactions. Furthermore, a small amount of unreacted CH was present in MK-CH; hence, the onset of mass loss in MK-CH occurred at a lower temperature, starting at approximately 500 °C. The mass loss of MK and MK-CS during this stage was primarily caused by type Ⅰ CSH gel. The addition of CS had a minor effect on the type I CSH gel, so the difference in mass loss between the two was not significant at this stage.

### 3.7. Effects of Calcium Sources on Hydration Products Microstructure

**Figure 9:**
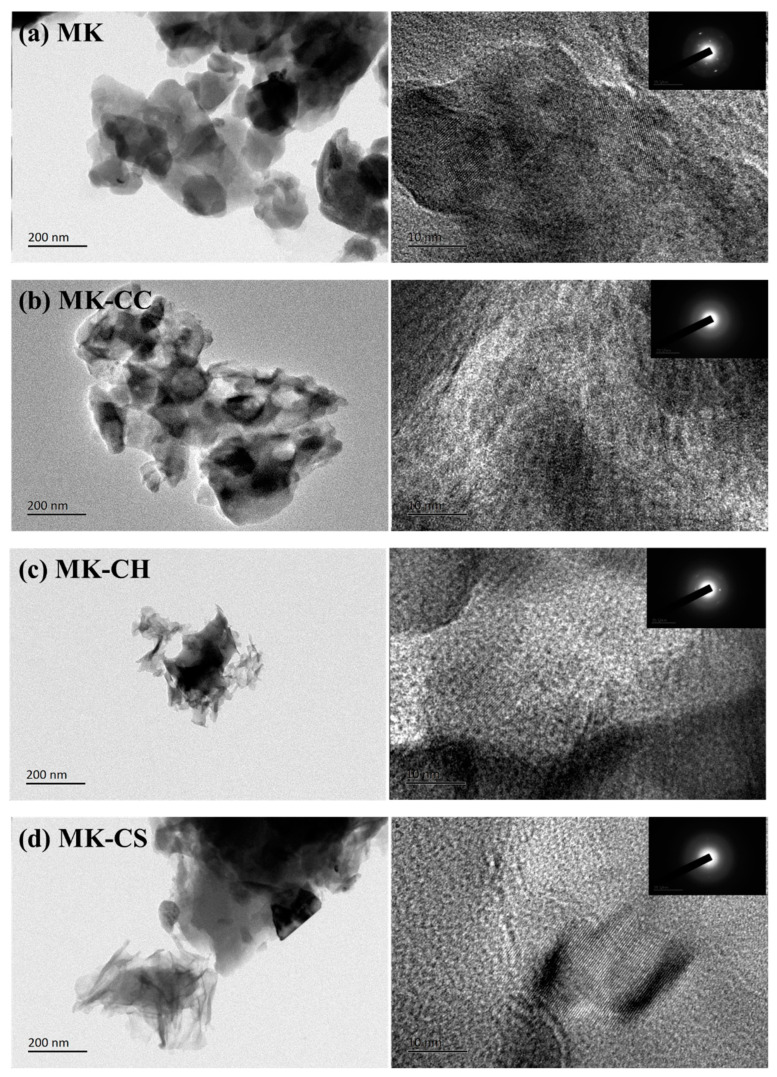
TEM micrographs (**left**) and electron diffraction (**right**) of the metakaolin geopolymers at curing age of 28 d.

**Figure 10:**
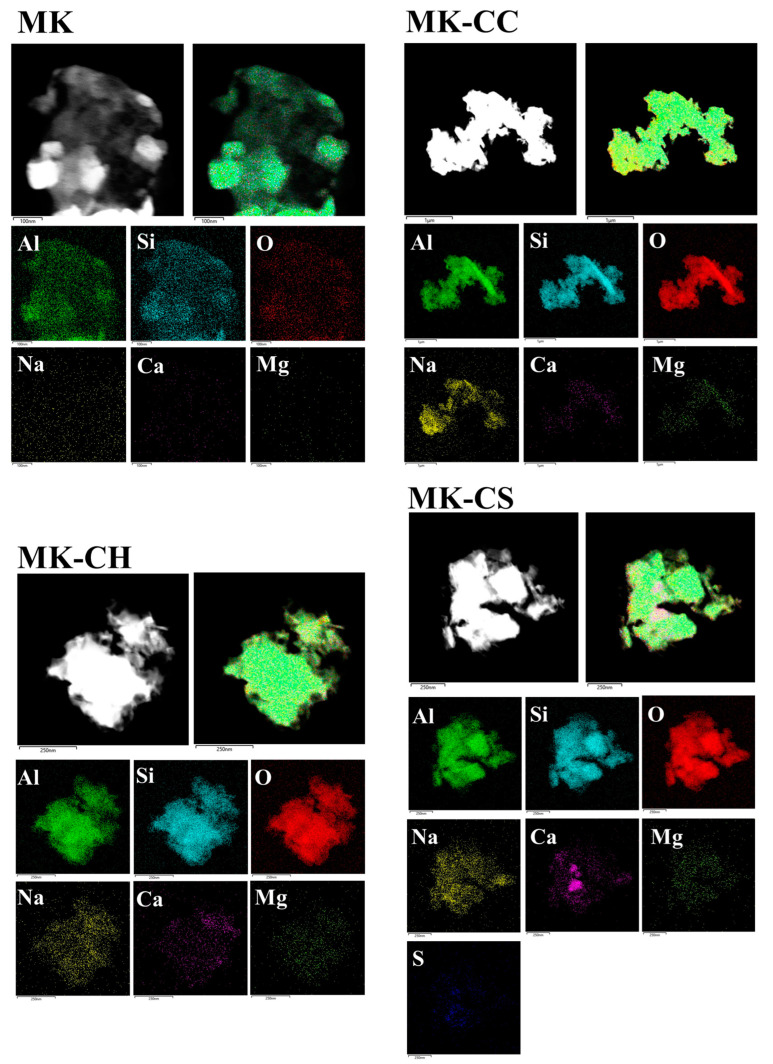
Element distribution images of the metakaolin geopolymers at curing age of 28 d.

Figure 9 and Figure 10 show the TEM images of hardened pastes after curing of 28 d. It was observed that the hardened paste primarily comprised NASH gel with a minor quantity of CASH gel. The NASH gel in MK was mainly cotton-like with a size of 100~200 nm [60]. Despite no addition of a calcium source, the presence of CASH gel was still detected in MK, attributed to the intrinsic small amount of CaO in MK. The NASH gels in MK-CC were also cotton-like with a size of 100~200 nm. The Ca content in the gels was very low from the aspect of element composition, indicating that calcium carbonate did not participate in the hydration reaction, which was consistent with the results of TG and FTIR. Through the magnification observation of NASH gels in MK and MK-CC, it was found that some crystals were contained in the amorphous regions. The diffraction spots also showed that the two gel structures were amorphous structures mixed with some microcrystals.

Two micro-morphologies of gels appeared in MK-CH and MK-CS, respectively: the foil-like structure of CASH and the cotton-like structure of NASH [61,62,63]. From the aspect of element composition, the density of Ca^2+^ was found to be higher in MK-CH and MK-CS, suggesting that the incorporation of CH and CS increased the Ca/Si ratio of the gel, confirming prior speculations. Through the high-resolution observation and diffraction analysis of the gels in MK-CH and MK-CS, it was found that the gels in the two samples were also amorphous structures mixed with some microcrystals. In addition, a relatively concentrated distribution of Ca and S had been observed in MK-CS, which could have resulted from unreacted CS or the formation of ettringite. Due to the relatively high content of MgO (as shown in Table 1) in MK, a uniform distribution of Mg across all geopolymers was observed, indicating the presence of a certain amount of MASH gel within geopolymers.

### 3.8. Prospect of Different Calcium Sources of Metakaolin Geopolymers

Calcium sources of different anions had different effects on the reaction process and hydration products of geopolymers. The pozzolanic reaction between CH and MK could promote MK hydration and increase the proportion of CASH gel in the hydration products, thereby facilitating the setting of the geopolymer and enhancing its strength. CS could react with the active aluminates in MK to form ettringite, thus forming a higher early strength, and a large number of new hydration products were formed in the system. CC had a lower reactivity with MK and did not improve the performance of geopolymers.

It is well known that CH, CS, and CC correspond to calcium carbide residue, desulfurization gypsum, and soda residue in high-calcium industrial solid waste, respectively. The use of these low-carbon calcium source solid wastes in actual geopolymer production could not only reduce production costs, but also improve mechanical properties. Through the research in this paper, it could be inferred that calcium carbide residue can be used as an excellent calcium source in geopolymer.

## 4. Conclusions

This study aimed to enhance the mechanical properties of MK-based geopolymer by introducing calcium sources. The effects of three different calcium sources, CH, CC, and CS, on the properties of the geopolymer were investigated, and their effect mechanisms were analyzed, leading to the following main conclusions:

1. The addition of CH and CS accelerated the setting and hardening of the MK-based geopolymer, with CH having the most significant effect, reducing the setting time of the geopolymer from 358 min to 44 min.
2. Apart from CC, both CH and CS were able to enhance the strength of the MK-based geopolymer; however, CH improved the strength at both 7 d and 28 d, while CS only significantly increased the strength at 7 d.
3. The pH value of the pore solution in the geopolymer also changed with the addition of calcium sources. For geopolymers with added CC and CH, the pH value of their pore solution decreased as the curing time increased, whereas for geopolymers with added CS, the pH value of their pore solution increased as the curing time increased.
4. The type of calcium source significantly affected the hydration heat-release behavior of MK-based geopolymers. The addition of CC reduced the heat-release rate and the total heat release of the geopolymer, while the inclusion of CH and CS increased both the heat release rate and the total heat release. After adding CH, the geopolymer exhibited a distinct peak in the heat-release rate between 5 and 12 h. Following the addition of CS, two peaks in the heat-release rate occurred, respectively, within 1–3 and 12–15 h.
5. The primary hydration product in MK-based geopolymers was NASH, and the addition of calcium sources significantly affected the hydration products. The addition of CC, CH, and CS promoted the formation of CASH gel within the geopolymer, with CH having the most significant effect. The addition of CS not only facilitated the formation of CASH gel, but also reacted with the active alumina in MK to form ettringite. CC had the least participation in the hydration reaction and contributed minimally to the content of CASH gel.

## Figures and Tables

**Table 1:** Chemical compositions of MK (wt. %).

Materials	CaO	SiO_2_	Al_2_O_3_	Fe_2_O_3_	MgO	Na_2_O	K_2_O	SO_3_
MK	0.62	55.54	40.61	1.08	0.32	0.07	0.88	0.05

**Table 2:** Modified geopolymer ratios (wt. %).

Sample	MK	CaCO_3_	Ca(OH)_2_	CaSO_4_	w/b
MK	100	-	-	-	0.5
MK-CC	80	20	-	-	0.5
MK-CH	80	-	20	-	0.5
MK-CS	80	-	-	20	0.5

**Table 3:** Final setting time of the metakaolin geopolymers.

Sample	Final Setting Time (min)
MK	358
MK-CC	604
MK-CH	44
MK-CS	137

## Data Availability

Data are contained within the article.
